# Alumina and glass-bead blasting effect on bond strength of zirconia using 10-methacryloyloxydecyl dihydrogen phosphate (MDP) containing self-adhesive resin cement and primers

**DOI:** 10.1038/s41598-023-46548-4

**Published:** 2023-11-05

**Authors:** Ahmed Abdou, Nasser Hussein, Citra Kusumasari, Emad A. Abo-Alazm, Amr Rizk

**Affiliations:** 1 Faculty of Dentistry, Al-Ayen University, Thi-Qar, Iraq; 2 Fixed Prosthodontics Department, Faculty of Dentistry, Modern University for Technology, and Information, Mokatam, Cairo, Egypt; 3 Department of Conservative Dentistry, Faculty of Dentistry, Universitas Indonesia, Jakarta, Indonesia; 4 Restorative Dentistry Department, Faculty of Dentistry, Egyptian Russian University, Badr City, Cairo, Egypt; 5 Prosthetic Dentistry Department, Fixed Prosthodontics Division, Faculty of Dentistry, King Salman International University, South Sinai, El Tur, 46511 Egypt

**Keywords:** Dental materials, Prosthetic dentistry, Restorative dentistry

## Abstract

In fact, bonding of zirconia restorations is still a big challenge in clinical situations and many bonding protocols discussed in literature might be still controversial. The aim of this was to study assess the bond strength of zirconia after alumina and glass-bead pre-treatments with two different primers in combination with conventional resin cement and 10-methacryloyloxydecyl dihydrogen phosphate (MDP) containing self-adhesive resin cement without priming. Fully sintered high translucent zirconia samples (*n* = 160) were assigned into 2 groups of pre-treatments (*n*  = 80): Alumina-sandblasting (AB) and Glass-bead (GB). Then, each group was divided into 4 sub-groups according to priming and cement used (*n*  = 20 each): conventional self-adhesive resin cement, MDP-silane Primer, MDP primer both with conventional self-adhesive resin cement, and MDP contained cement. Shear bond strength (SBS) was measured after thermocycling. Failure mode was analyzed using stereomicroscope. Contact angle and surface topography were investigated using other fully sintered samples (*n*  = 30) constructed for that sole purpose, divided into control (no pre-treatment [unmodified], alumina-, and glass-bead sandblasted groups). Two-way ANOVA was performed for SBS and failure mode was analyzed. The use of Alumina-sandblasting showed higher SBS compared to Glass-bead pre-treatment for MDP-silane primer (*p* = 0.034) and MDP primer (*p* < 0.001). While MDP contained cement showed higher but insignificant SBS when pre-treated with glass-beads. Alumina-sandblasting and glass-bead pre-treatments improve bond strength of zirconia combined using primers before cementation with conventional resin cement. Also, self-adhesive MDP contained cement along with surface pre-treatment showed the highest achievable bond strength. It was concluded that both alumina-sandblasting and glass-bead blasting improved SBS combined with MDP containing self-adhesive resin cement reducing the required clinical steps during cementation of zirconia restorations.

## Introduction

Nowadays, the demand for highly esthetic restorations is increasing in everyday dental practice owing to the fast-paced evolvement and innovation in digital dentistry. Dental zirconia is considered one of the most commonly used restorative materials due to its high mechanical properties compared to glass ceramics^1^. That leads to increase the range of indications in the field of fixed prosthodontics and indirect restorations, but earlier zirconia generations suffered from lower translucency, higher opacity and hence, less superior esthetics as it is composed of dense polycrystalline structure with no glass matrix when compared to glass ceramics limiting its use to posterior region^2^. To improve the optical properties, increasing the yttria content to 5 mol% yttria-stabilized tetragonal zirconia polycrystals (5Y-TZP) resulting in 50% cubic phase compared to conventional 3 mol% yttria stabilized tetragonal zirconia^3–5^ thus improving the overall aesthetics and resulted in the introduction of new class of ultra-translucent zirconia.

The bonding of zirconia to tooth tissues or other synthetic materials is controversial when compared to silica-based ceramic material due to its chemical inertness and resistance to aggressive chemical agents (strong acid, alkalis, organic and inorganic dissolving agents^6^. The incidence of loss of retention for zirconia restorations was 4.7% over the 5-year observation period^7^, and it is the directly caused by the deboning between the various adhesive interfaces within that structure^8^.

There are many ways for increasing the bond strength between zirconia ceramic and resin cement had been investigated in literature, either mechanical, chemical, and/or chemico-mechanical surface pre-treatment methods^9–11^. Two published meta-analyses have confirmed the importance of the combined mechano-chemical surface treatment for improving the bond strength of zirconia with resin cements^12, 13^. Mechanically, air-borne-particle abrasion, tribochemical airborne-particle abrasion, low-fusion porcelain application, hot chemical etching solutions, selective infiltration etching, laser irradiation, plasma spraying, and zirconia ceramic powder coating were proposed, while zirconia primers with 10-Methacryloyloxydecyl dihydrogen phosphate (10-MDP) molecule and its salt were proposed to alter the zirconia surface chemistry^9, 14–17^.

The air abrasion method with alumina particles showed the highest bond strength in comparison with other surface conditioning methods^18^. However, that alumina air abrasion produces surface defects such as flaws, plastic deformation, embedded abrasive alumina, and microcracks, which can compromise the mechanical properties of zirconia and decrease fracture strength^19^. To decrease the defect to zirconia surface, the use of glass beads as a softer material than sharp and hard alumina was suggested by Khanlar et al., in 2022, followed by 10-MDP & silane primer which reported a desirable bonding performance without creating surface microcracks on zirconia^20^.

There are scarce reports on the effect of glass-beads blasting as a mechanical bonding method either alone or combined chemically with different zirconia primers. Hence, this in vitro study was conducted to evaluate the effect of alumina-sandblasting and glass-bead blasting on bond strength of high translucent (5Y-TZP) zirconia solely or combined with different formulations of zirconia primers and MDP contained self-adhesive resin cement without subsequent priming. The null hypotheses tested were that 1. sandblasted zirconia either with alumina- and/or glass-beads will show similar bond strength, 2. different MDP-primers will not affect the bond strength to zirconia 3. MDP containing self-adhesive resin cement will show a similar result to other MDP-primers tested.

## Materials and methods

### Materials

The tested substrate is a high translucent yttria-stabilized tetragonal zirconia polycrystals specimens (5Y-PSZ; Liaoning Upcera Co., Ltd. Liaoning, China). An MDP-Silane primer (Visalys retoration primer, Kettenbach GmbH & Co. KG, Eschenburg, Germany) and an MDP-BPDM primer (Z-prim, Bisco Inc., IL, USA) were used. For cementation, conventional resin cement (Visalys CemCore, Kettenbach GmbH & Co. KG) and self-adhesive resin cement (Z-prim, Bisco Inc.) were used in the current study. The full compositions of used materials are listed in Table 1.

**Table 1:** Materials used in the current study.

Material	Manufacturer	Composition [Batch]
TT/ML Top translucent/Multi-layer	Liaoning Upcera Co., Ltd. Liaoning, China	86.3–94.2 wt% ZrO_2_ + HfO_2_, 5.8–9.7 wt% Y_2_O_3_, < 0.5 wt% Al_2_O_3_, < 2.0 wt% Er_2_O_3_, < 0.5 wt% other oxides [L2190905167-48]
Alumina (Al_2_O_3_)	Kulzer,Japan	Al_2_O_3_ 50 µm
Glass bead air abrasion	Shofu Inc., Koyoto, Japan	Soda-lime glass beads SiO_2_-R_2_O_3_-R_2_O-RO (R_2_O_3_: Al_2_O_3_) (R_2_O: Na_2_O, K_2_O) (RO: CaO, MgO)
Visalys restorative primer [MDP + Silane]	Kettenbach GmbH & Co. KG, Eschenburg, Germany	10-MDP, Silane coupling agent, Ethanol [210151]
Z-prime plus [MDP + BPDM]	Bisco Inc., IL, USA	10-MDP, BPDM (Bis-GMA, HEMA) Ethanol [2100007462]
Visalys CemCore [conventional resin cement]	Kettenbach GmbH & Co. KG, Eschenburg, Germany	UDMA, other Dimethacrylate (aliphatic Trimethacrylate / aliphatic Dimethacrylate), Ytterbium fluoride and silica Polymorph, Benzoyl peroxide [210331006]

10-MDP; 10-methacryloyloxydecyl dihydrogen phosphate. BPDM; Bisphenyl dimethacrylate. HEMA; 2-Hydroxyethyl methacrylate monomethyl ether. Bis-GMA; Bisphenol A di (2-hydroxy propoxy) dimethacrylate. UDMA; Urethane dimethacrylate.

### Samples preparation and grouping

A total of 160 squares samples of 5Y-PSZ (10 × 10 × 3 mm before sintering) using a low-speed precision saw machine (Isomet 4000, Buehler, Lake Bluff, IL, USA) in a dry condition without water coolant. Another 160 disc shaped samples of a 4 mm dimeter and 3 mm thickness were used to be cemented to each other for shear bond strength evaluation. For surface roughness and contact angle measurements, another 30 square samples with previously mentioned dimensions were utilized. All samples were sintered utilizing a 7-stages cycle at 1450℃ following the manufacturer instruction in zirconia furnace (Lindberg/Blue M, Asheville, NC, USA). Sintering process parameters are listed in Table 2.

**Table 2:** Sintering process parameters.

Stages	Initial Temp (℃)	Final Temp (℃)	Time (min)	Heating Rate (℃ / min)
Stage 1	100	1150	131.25	8
Stage 2	1150	1150	30	0
Stage 3	1150	1300	75	2
Stage 4	1300	1450	37.5	4
Stage 5	1450	1450	120	0
Stage 6	1450	800	81.25	-8
Stage 7	800	150		Natural cooling

Sintering cycle total time: 7 h 55 min without natural cooling time.

**Figure 1:**
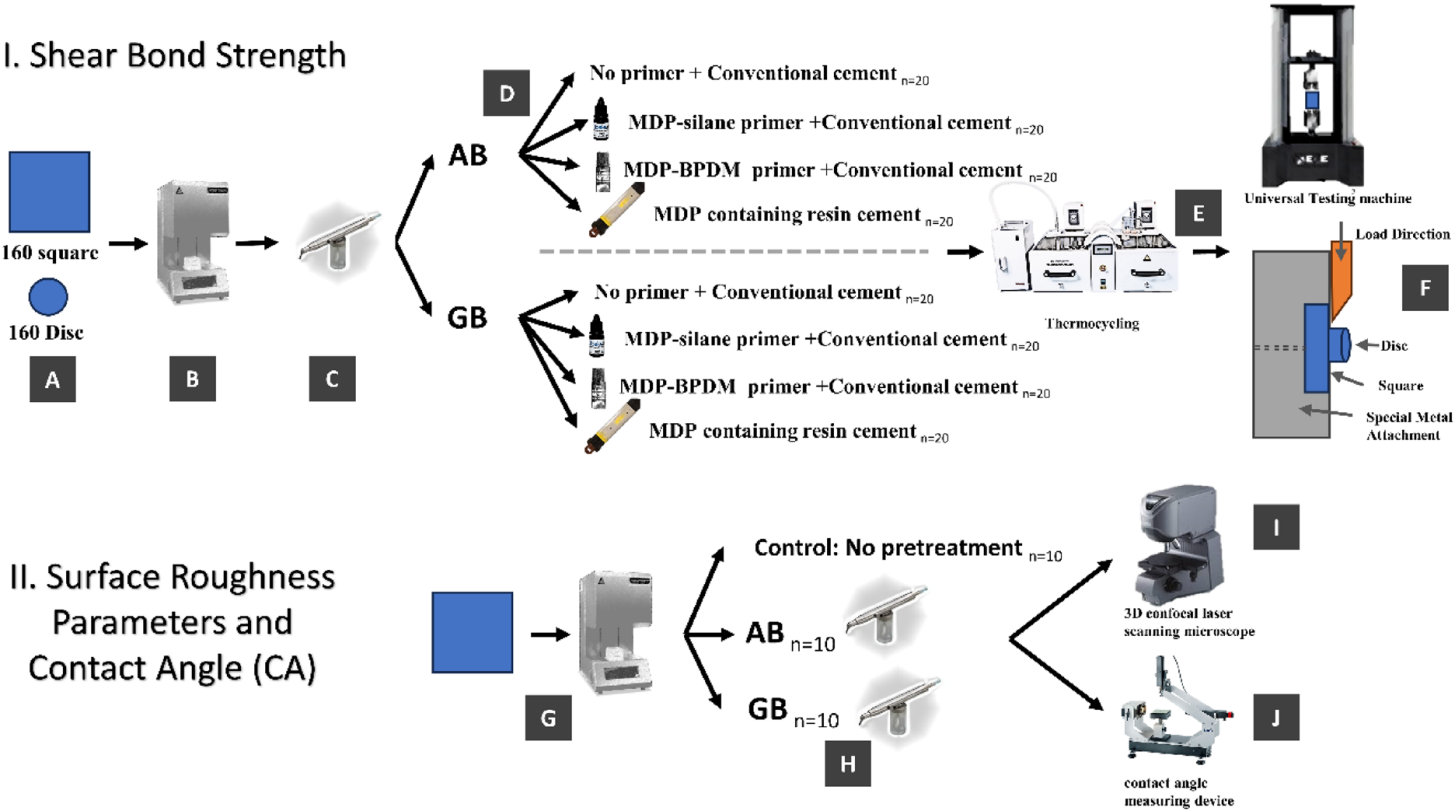
Schematic represntation of the methdology implanted in the curunt reserch. (A – F) represnets the steps for shear bond strength testing. A: 160 Square and 160 disc shaped samples prepared. B:Sinitring was done according to Table 2. C: Pre-treatment with Alumina-sandblasting (AB) and Glass-beads (GB). D: Each group was sub-divided into 4 sub-groups according to the cementation protocol (*n*  = 20 each); 1. no priming + conventional resin cement, 2. MDP-silane primer + conventional resin cement, 3. MDP-BPDM primer + conventional resin cement and 4. MDP containing resin cement without subsequent priming. E: After cementation, thermocycling for 10,000 cycle. F.Samples attached to universial testiong machine accodring to schamtic illustartion for Shear bond strentght. (G – J) represnts the surface parmater and contact angle measurments. G:30 square samples were prepaperd and sintered according to Table 2 steps. H: Samples were dividedinto three groups; control (no pre-treatment), Alumina-sandblasting (AB), and Glass-beads (GB). I: Sample were tested using 3D-CLSM for surface roughness parmaters. J: Sample were tested using contact angle measuring device.

For shear bond strength analysis, the fully sintered samples [both square and disc shaped (*n* = 160 each)] were divided equally into two groups according to the pre-treatment protocol (*n*  = 80 each); Alumina-sandblasting (AB) and Glass-beads (GB) groups. Each group was further sub-divided into 4 sub-groups according to the cementation protocol (*n*  = 20 each); 1. no priming + conventional resin cement, 2. MDP-silane primer + conventional resin cement, 3. MDP-BPDM primer + conventional resin cement and 4. MDP containing resin cement without subsequent priming. A graphical presentation of the methodology is presented in Fig. 1.

### Bonding protocols

A 600-grit silicon carbide paper was used to polish all samples before cementation. AB and GB groups were blasted with 50 µm Al_2_O_3_ (Kulzer, GmbH, Germany) and 75 µm Glass-beads particles respectively. The blasting time for both groups were 20 s at a pressure of 25 psi at a 10 mm distance and 90° angle using a sandblasting device (Microetcher IIA, Danville Materials, SanRamon, CA, USA). Afterwards, all samples were cleaned using ultrasonic cleaner (Easyclean MD, Renfert, GmbH, Germany) filled with distilled water for two minutes and then completely dried with oil-free air.

Each large square-shaped sample was bonded to the smaller disc-shaped sample. For the conventional resin cement groups, a dual cured resin cement (Visalys Cem-Core, Kettenbach GmbH & Co. KG, Eschenburg, Germany) was applied after alumina & glass-beads sandblasting respective to each group with no prior priming. For MDP-silane primer group: MDP-Silane primer (Visalys Restorative Primer, Kettenbach GmbH & Co. KG, Eschenburg, Germany) was applied by a micro-brush and left to dry for 60 s and air dried, then conventional resin cement was applied. For MDP-BPDM primer groups, samples were primed with 2 coats of MDP-BPDM primer (Z-Prime Plus, BISCO Inc., IL, USA) for 5 s then gentle air jet was applied followed by conventional resin cement. For MDP-containing self-adhesive resin cement group, all samples were cemented with dual-cured MDP-containing self-adhesive resin cement (TheraCem, Bisco Inc., IL, USA) with no prior priming. A static standard load of 5 kg was applied to all samples during cementation with the aid of a loading device^12^ and cement curing were initiated using LED light with a curing intensity of 1500 mW /cm^2^ (Eighteen CuringPen, Sifary medical technologies, Jiangsu Province, China) for 40 s.

### Shear Bond Strength (SBS) testing and failure mode analysis

All samples were aged by the aid of a thermocycler (SD Mechatronik, Germany) to induce hydro-thermal stresses within the tested material and the cement interface. The bonded samples were subjected to 10,000 cycles by immersing in distilled water bath of 5 °C and 55 °C with a dwell time of 20 s and a lag time of 10 s simulating one year of clinical service in the oral cavity^13^. A universal testing machine (Instron, model 3345, England) was used to measure and record the shear bond strength values with a crosshead speed of 1 mm/min. For the mode of failure analysis, a stereomicroscope (Olympus, Tokyo, Japan) was used at a magnification of 20 × to examine the de-bonded interface and failure was categorized as; adhesive failure “A”, cohesive failure “C” and mixed failure “M”.

### Surface topography parameters

For surface roughness, 30 square fully sintered samples were divided into 3 groups according to type of pre-treatment (*n* = 10 each), into control group (no alumina or glass beads sandblasting), Alumina-sandblasting (AB) and Glass-beads (GB).The samples were analyzed using a 3D confocal laser scanning microscope (CLSM, Keyence VK-X100, Keyence, Japan) with a 50 × magnification (scanning area 205 × 273.3 μm) and a MultiFile Analyzer software (V.1.3.1.120, Keyence) was used to analyze obtained scans. Arithmetical mean height (Sa), developed interfacial area ratio (Sdr) and texture aspect ratio (Str) values were recorded and analyzed.

### Contact angle measurements

After measuring surface roughness parameters, the same samples were used to measure the surface wettability adopting sessile drop method using deionized water by the aid of a contact angle measuring device (DSA25B, Krüss GmbH, Germany). For each sample, 3 readings were recorded for 3 different drops and the average value was considered the mean reading for every tested sample. All mean values were reported for statistical analysis.

### Statistical analysis

Sample size was calculated based on data extracted from Khanlar et. al. 2022^20^. A minimum of 20 samples in each group will be sufficient to detect a power of 95% when α = 0.05. The mean for control was 9.2 and for AB was 11.7 with a standard deviation equals to 2 resulted in an effect size (d) of 1.2. Sample size was calculated using G*Power version 3.1.9. Data explored for normality using Shapiro–Wilk test. Two-way ANOVA used to compare between tested pre-treatment and primer/cement followed by Tukey’s HSD test for pairwise comparison. Additional, shear bond strength data were analyzed using Weibull analysis (R4, R Foundation for Statistical Computing, Vienna, Austria). Weibull parameters were calculated using Wald estimation, and 95% confidence intervals were calculated with Monte Carlo simulations. The different groups were compared at the characteristic strength (63.2% and 10% probability of failure). For surface roughness parameters and contact angle measurements, one-way ANOVA used to compare between tested groups followed by Tukey’s HSD test for pairwise comparison. (α = 0.05). Statistical analysis was performed with IBM SPSS Statistics for Windows, Version 26.0. Armonk, NY: IBM Corp.

## Results

### Shear bond strength (SBS)

Pre-treatment and primer/cement groups resulted in significant effect on shear bond strength at *p* = 0.003 and *p* < 0.001, respectively. The interaction between both variables resulted in a significant effect on shear bond strength at *p* = 0.002 as shown in Table 3. Results of pairwise comparison showed that, alumina sandblasting (AB) showed higher shear bond strength compared to glass beads (GB) for MDP-silane (*p* = 0.034) and MDP-BPDM (*p* < 0.001) groups. While for both conventional resin cement groups and MDP containing self-adhesive resin cement showed insignificant difference when pre-treated with AB and GB. For AB pre-treated groups, conventional resin cement showed the lowest significant shear bond strength compared to all other groups and insignificant difference resulted between MDP-silane primer, MDP-BPDM primer, and MDP containing cement. For GB pre-treated groups, conventional resin cement showed the lowest significant shear bond strength and MDP containing resin cement showed the highest shear bond strength compared to all other groups. Insignificant difference between MDP-silane primer and MDP-BPDM primer. Shear bond strength values for different tested groups are presented in Table 4.

**Table 3:** Two-Way ANOVA for the effect of different pre-treatment and primer/cement on the mean shear bond strength.

Source	Type III Sum of Squares	df	Mean Square	F	Sig
Pre-treatment	646.770	1	646.770	9.659	0.003
Primer	8609.485	3	2869.828	42.858	< 0.001
Pre-treatment * Primer	1064.167	3	354.722	5.297	0.002

**Table 4:** Mean and SD for Shear bond strength in MPa for different tested groups.

Primer/cement	AB		GB		*p*-value
	Mean	SD	Mean	SD	
Conventional resin cement	10.54^a^	2.46	5.79^a^	3.07	0.301
MDP-Silane Primer + Conventional resin cement	29.49^b^	8.84	21.74^b^	7.96	0.034
MDP-BPDM primer + Conventional resin cement	35.84^b^	5.83	20.17^b^	6.81	< 0.001
MDP contained resin cement	38.64^b^	14.45	42.93^c^	6.96	0.235
*p*-value	< 0.001*		< 0.001*		

Different letters within each column indicate significant difference. Significant level was set at p < 0.05 (adjusted Tukey’s HSD).

**Figure 2:**
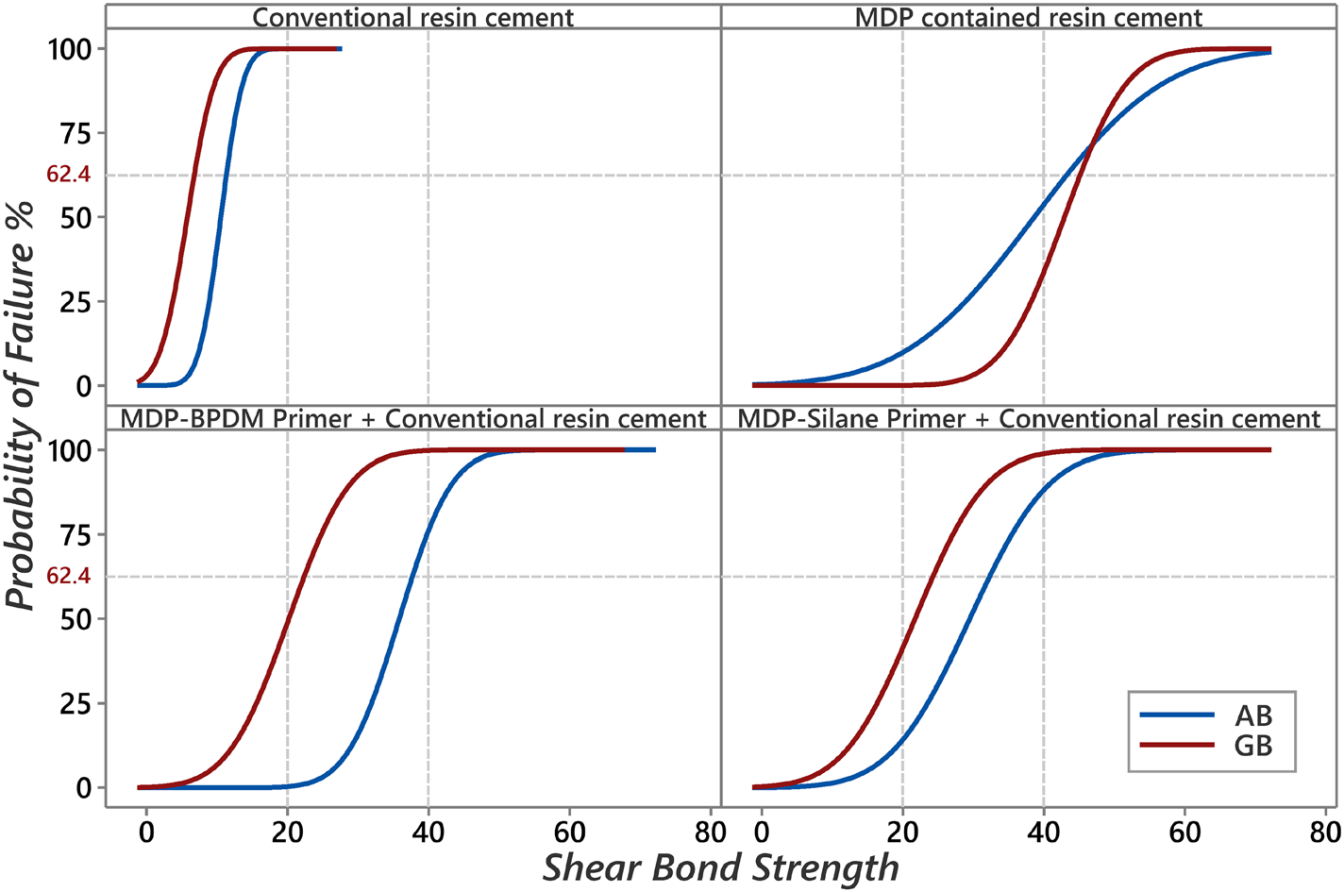
TThe Weibull survival graphs of the shear bond strength (MPa) of the tested groups. A horizontal dashed line at 63.2% probability of failure helps to compare the characteristic strengths. Vertical reference dashed line at 20 MPa and 40 MPa for comparing survival curves of the tested groups. MDP containing resin cement showed the highest characteristic strength compared to all other primers/cement protocol.

Weibull analysis results presented in Table 5 and Fig. 2. The Weibull characteristic strength for conventional resin cement was significantly lower than all other groups primer/cement with AB and GB within insignificant difference between each other’s. For AB pre-treatment, an insignificant difference between MDP-Silane + Conventional cement, MDP-BPDM + Conventional cement, and MDP containing resin cement resulted for the Weibull characteristic strength. For GB pre-treatment, MDP containing resin cement showed the significantly highest Weibull characteristic strength compared to all other groups. AB + MDP-BPDM + Conventional cement and GB + MDP containing resin cement showed the highest Weibull modulus. Both ANOVA and Weibull analysis showed a similar results except that AB and GB were not signifcantley different in case of MDP-Silane Primer + conventional cement in case of Weibull analysis.

**Table 5:** Weibull analysis and failure mode for tested groups.

Surface pretreatment	Primer/cement	α [95% CI]	β [95% CI]	P10 [95% CI]	FM [A/M/C]
AB	Conventional resin cement	11.47[9.84 to 13.37]^a^	5.12[3.24 to 12.14]	7.39[5.54 to 10.36]^b^	[60/40/0]
	MDP-Silane Primer + Conventional resin cement	32.65[27.32 to 39.03]^cd^	3.69[2.53 to 6.81]	17.74[12.14 to 25.91]^cd^	[90/10/0]
	MDP-BPDM primer + Conventional resin cement	38.2[35.11 to 41.57]^d^	7.74[5.14 to 15.68]	28.56[23.59 to 34.59]^d^	[60/40/0]
	MDP contained resin cement	42.65[34.85 to 52.19]^de^	3.16[2.04 to 6.98]	20.91[12.95 to 33.78]^cd^	[20/80/0]
GB	Conventional resin cement	6.57[4.5 to 9.59]^a^	2.23[1.37 to 6.04]	2.40[1.04 to 5.27]^a^	[100/0/0]
	MDP-Silane Primer + Conventional resin cement	24.32[19.87 to 29.77]^bc^	3.09[2.13 to 5.6]	11.74[7.59 to 18.15]^bc^	[100/0/0]
	MDP-BPDM primer + Conventional resin cement	22.47[18.83 to 26.81]^b^	3.52[2.39 to 6.71]	11.85[7.97 to 17.62]^bc^	[100/0/0]
	MDP contained resin cement	45.8[41.93 to 50.03]^e^	7.09[4.88 to 12.91]	33.34[27.53 to 40.37]^d^	[100/0/0]

Different superscript letters within the α and P10 columns are statistically significant differences based on a 95% confidence interval (CI). α: characteristic strength or scale of a Weibull parameter. β: the shape, slope, modulus of a Weibull parameter. P10: estimation at 10% probability of failure. FM: failure modes percentage; (A) adhesive failure at ceramic-resin interface, (C) cohesive failure at the resin cement, and (M) mixed failure.

### Failure mode

**Figure 3:**
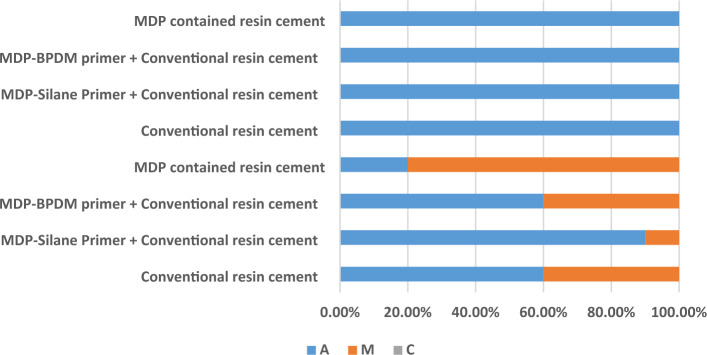
Staked Bar chart showing the failure mode for different tested groups. **A** Adhesive failure, (M) Mixed failure, and **C** Cohesive failure.

For glass beads (GB) pre-treatment, all the tested groups showed 100% adhesive failure. While for alumina sandblasting (AB) pre-treatment, all groups showed both mixed failure and adhesive failure. No group showed cohesive failure for both GB and AB. The mode of failure analysis is represented in Fig. 3 and a representative image for failure mode are presented in supplementary file.

### Surface roughness parameters and contact angle (CA)

**Figure 4:**
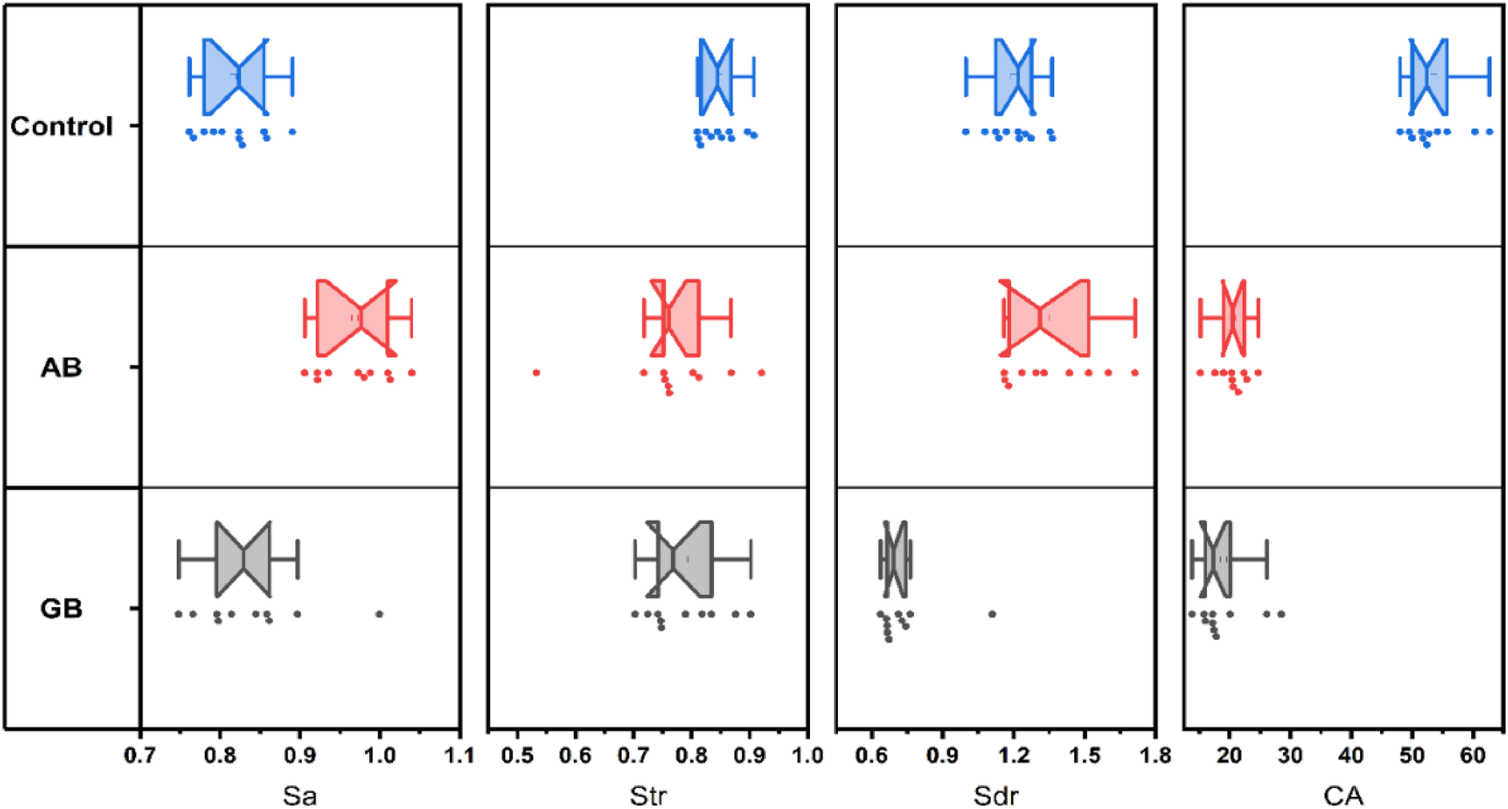
Box plot showing the surface topography parameters [Arithmetical mean height (Sa), developed interfacial area ratio (Sdr), and texture aspect ratio (Str)]and contact angle (CA) measurements for different tested groups.

Results of surface roughness parameters and contact angle are presented in Fig. 4. For Sa, Control group (0.82 ± 0.04) and GB (0.84 ± 0.07) showed the lowest significant Sa values compared AB (0.97 ± 0.05) at *p* < 0.001. While for Sdr, Control group (1.20 ± 0.11) and AB (1.36 ± 0.70) showed the highest significant Sdr values compared GB (0.74 ± 0.14) at *p* < 0.001. For Str, Control group (0.85 ± 0.03) showed the lowest significant str values compared AB (0.77 ± 0.10) at *p* = 0.044. GB (0.79 ± 0.07) showed insignificant difference with both control and AB for Str values. Contact angle measurements showed that control group (50.53 ± 4.29) had the highest significant values compared AB (44.94 ± 2.12) at *p* = 0.038. GB (47.60 ± 2.34) showed insignificant difference with both control and AB for contact angle measurement.

## Discussion

Over the last decades zirconia was introduced in dentistry to be used as a stronger alternative replacing weaker silica-based ceramics and increasing the range of its indications as a restorative alternative. However, bonding to zirconia has always been a major problem even with the development of more translucent monolithic alternatives since most of bonding protocols are based upon bonding to the glass matrix that can be eroded by the effect of a strong acid which is not the case with zirconia as it lacks the presence of the glassy matrix due to its polycrystalline nature.

This study investigated the bond strength to zirconia after alumina and glass-bead blasting with two different MDP-primers in combination with conventional resin cement, in addition to an MDP containing self-adhesive resin cement without priming. The tested null hypotheses were rejected as alumina- and glass-beads sandblasting combined with priming before cementation and MDP contained cement used solely showed higher bond strength when compared to sandblasted groups that were cemented with conventional resin cement with no priming.

The bond strength created between zirconia and the adhesive cement plays a major role in the success and longevity of dental restorations. A weak adhesion might be a major cause in crack formation in the restorative material that may reach the cement interface causing failure of a restoration^21^.

The results of this study revealed that alumina or glass-beads sandblasting combined with chemical surface treatments (primer) resulted in a significant increase in shear bond strength of 5Y-PSZ zirconia. In previous studies, it was found that air abrasion with alumina particles is the most preferred and reliable surface treatment method for high strength ceramics as it increases surface roughness resulting in an in-creased surface energy, improving wettability, and may decontaminate bonding surfaces^22, 23^. As for glass-beads the increase of bond strength may be attributed to the incorporation of silica to the bonded surface resulting in stable chemical bonds between the hydroxyl groups (OH) of the silica of the glass surface and the primer/resin cement^24^. Another study evaluated the effect of different silicatization protocols (Glass-beads and tribochemical coating) with various silane treatment methods on bond strength of translucent zirconia and they found that both alumina and glass-beads air blasting improved bond strength. Their finding was supported by Energy Dispersive Spectroscopy (EDS) and X-ray Photoelectron Spectroscopy (XPS)analysis which confirmed the deposition high silica content at the surface of cemented samples even that was not washed out by ethanol or ultrasonic cleaning^10^.

Surface roughness of the cemented zirconia may play a role in the adhesion as it may increase surface area of the cemented substrate. The surface roughness after alumina-blasting was higher compared to glass-beads blasted surface. The findings of this study were in agreement with Khanlar et al.^20^, who revealed that air abrasion with alumina particles had an effect of increasing the surface roughness while glass-beads had no effect. Moreover, their SEM/EDS investigations found that alumina created grooves and surface flowers while glass-beads resulted in the deposition of silica particles without affecting the surface roughness^20^. On the other hand, Mehari et al.^25^, found that alumina increased the bond strength while glass-beads and no treatment showed almost the same results for three different types of zirconia which may be attributed to the increased surface roughness to alumina blasted surface.

Alumina sandblasting showed the lowest significant contact angle and the highest surface roughness when compared to glass-beads and control group, both factors that led to high bond strength as per outcomes of our study. Translucent zirconia has larger grains size resulting in grains being pulled out readily during the alumina sandblasting causing surface defects and in succession increased zirconia surface roughness^26^. Moreover, alumina sandblasting led to formation of micro-mechanical means in terms of increased roughness enhancing surface energy and more resin flow in those micro-retentive features resulting in higher bond strength^27^. In previous studies, it was suggested that sandblasting may be responsible for generating hydroxyl groups on the zirconia surfaces, leading to the increase of zirconia reactivity with phosphate monomers in MDP affecting bond strength^28, 29^. This was not the situation with glass-beads, as the low hardness nature of glass-bead cannot alter surface roughness of zirconia. Instead, its effect was limited embedding the surface of zirconia with silica particles resulting in decreased contact angle and enhanced surface energy which improve the bonding^10, 20^.

The results of this study regardless of the pre-treatment method showed that MDP containing self-adhesive resin cement had the highest bond strength regardless of the pre-treatment method utilized followed by MDP-BPDM primer followed by MDP-silane primer for alumina air abrasion. As for groups with glass-beads pre-treatment, MDP-silane and MDP-BPDM containing primers had a similar bond strength value. Pure MDP results in better bonding performance to zirconia surface and addition of silane in one-bottle with MDP can decrease the bonding^16^. However, Pure-MDP primers are not available in the market and the commercially available primers combine more than one primer for universal application and compatibility with various substrates.

The phosphate easter groups in the MDP molecule in theory react with one or two zirconium atoms, forming two bonding configurations either “double coordinate” or “single coordinate”^30^. MDP containing primers has a hydrophobic phosphoric group that reacts with the hydroxyl groups on the surface of the translucent zirconia enhancing bond strength^31^. Additionally, MDP prevents the penetration of water between the hydrophobic phosphate layer and the oxide layer of zirconia by the action of decyl group in MDP^32^.

According to the literature, the presence of MDP in the resin luting agent forms a stable bond to airborne pre-treated zirconia even after thermocycling^33^. This may be attributed to the fact that MDP contains both a polymerizable methacrylate terminal end that adheres to resin and a hydrophilic phosphate terminal end that chemically adheres to zirconia enhancing the bond strength^21^.

The use of MDP-silane containing primer increased the bond strength between resin cement and zirconia ceramics as per findings in previous studies^15, 34, 35^. Also, with GB group the higher bond strength values are justified by chemical interaction between the silane and silica from the glass beads remaining on the zirconia surface^20^. However, the bond strength was lower than that of MDP containing self-adhesive resin cement. This may be attributed to silanols in the MDP-silane containing primer, which may result in that decrease in bond strength between MDP and zirconia surface^11, 36^.

In previous studies, it was found that MDP-BPDM containing primer had a positive effect on bond strength of cemented zirconia^37–39^. A previous study disagreed with our study finding, attributing the reason to the presence of carboxylic acid monomer in BPDM that may have a determinantal effect on the connection between this primer and self-adhesive resin cement methacrylate^40^.

Thus, based on that study the use of alumina air abrasion was always considered as the golden standard for bonding to zirconia, it worthy to mention that glass-beads can offer a promising alternative to enhance bonding to zirconia both in conjunction with a primer or MDP cement contained. From the limitations of this study, masticatory forces and anatomical fixed partial denture designs should be valuated to mimic the oral condition rather than the simplified design implanted in the current work. Additionally, further in-vivo studies are required to evaluate the efficacy of the proposed bonding protocols.

## Conclusion

Within the limitations of the current study and based on the findings, the following can be concluded:

1. Alumina-sandblasting and glass-bead pre-treatments improve bond strength of zirconia using MDP-primers or with MDP containing self-adhesive resin cement solely without prior priming.

2. MDP containing self-adhesive resin with no prior priming can be used as a successful cementation protocol with less clinical steps for successful zirconia cementation.

3. Sandblasting should be combined with a proper chemical treatment strategy to enhance bond strength to zirconia.

### Supplementary Information

Supplementary Information.

## Data Availability

Dataset used and analyzed data can be available form corresponding author on reasonable request.
