# Supplementary material for: Alumina and glass-bead blasting effect on bond strength of zirconia using 10-methacryloyloxydecyl dihydrogen phosphate (MDP) containing self-adhesive resin cement and primers
Source: Sci Rep. 2023 Nov 5;13:19127. doi: 10.1038/s41598-023-46548-4 (PMC10625984; doi:10.1038/s41598-023-46548-4)
Supplement: Supplementary file 1 — Supplementary Information. [file 41598_2023_46548_MOESM1_ESM.docx]

**Title: Alumina and Glass-bead Blasting effect on Bond Strength of Zirconia using 10-methacryloyloxydecyl dihydrogen phosphate (MDP) containing Self-adhesive Resin Cement and Primers**

Ahmed Abdou^1^, Nasser Hussein^2^, Citra Kusumasari^3*^, Emad A. Abo-Alazm ^4^, Amr Rizk ^5^

^1^ Faculty of Dentistry, Al-Ayen University, Thi-Qar, Iraq; Email:abdou.biomat@gmail.com.

^2^Fixed Prosthodontics Department, Faculty of Dentistry, Modern University for Technology, and Information, Mokatam, Cairo, Egypt.

^3^ Department of Conservative Dentistry, Faculty of Dentistry, Universitas Indonesia, Jakarta, Indonesia. Email: citra.kusuma02@ui.ac.id

^4^ Restorative Dentistry Department, Faculty of Dentistry, Egyptian Russian University, Badr City, Cairo, Egypt.

^5^ Prosthetic Dentistry Department, Fixed prosthodontics Division, Faculty of Dentistry, King Salman International University, El Tur, South Sinai, 46511, Egypt; email: amr.rizk@ksiu.edu.eg.

***Corresponding author-** Citra Kusumasari

Department of Conservative Dentistry, Faculty of Dentistry, Universitas Indonesia, Jakarta, Indonesia. Email: citra.kusuma02@ui.ac.id.

Phone No: 0062-816-1828-528

**Keywords:** zirconia primer; 10-methacryloxydecyl dihydrogen phosphate; alumina-sandblasting, glass-beads; self-adhesive resin cement, Wettability

**Supplementary file**

**Failure mode analysis:**

**
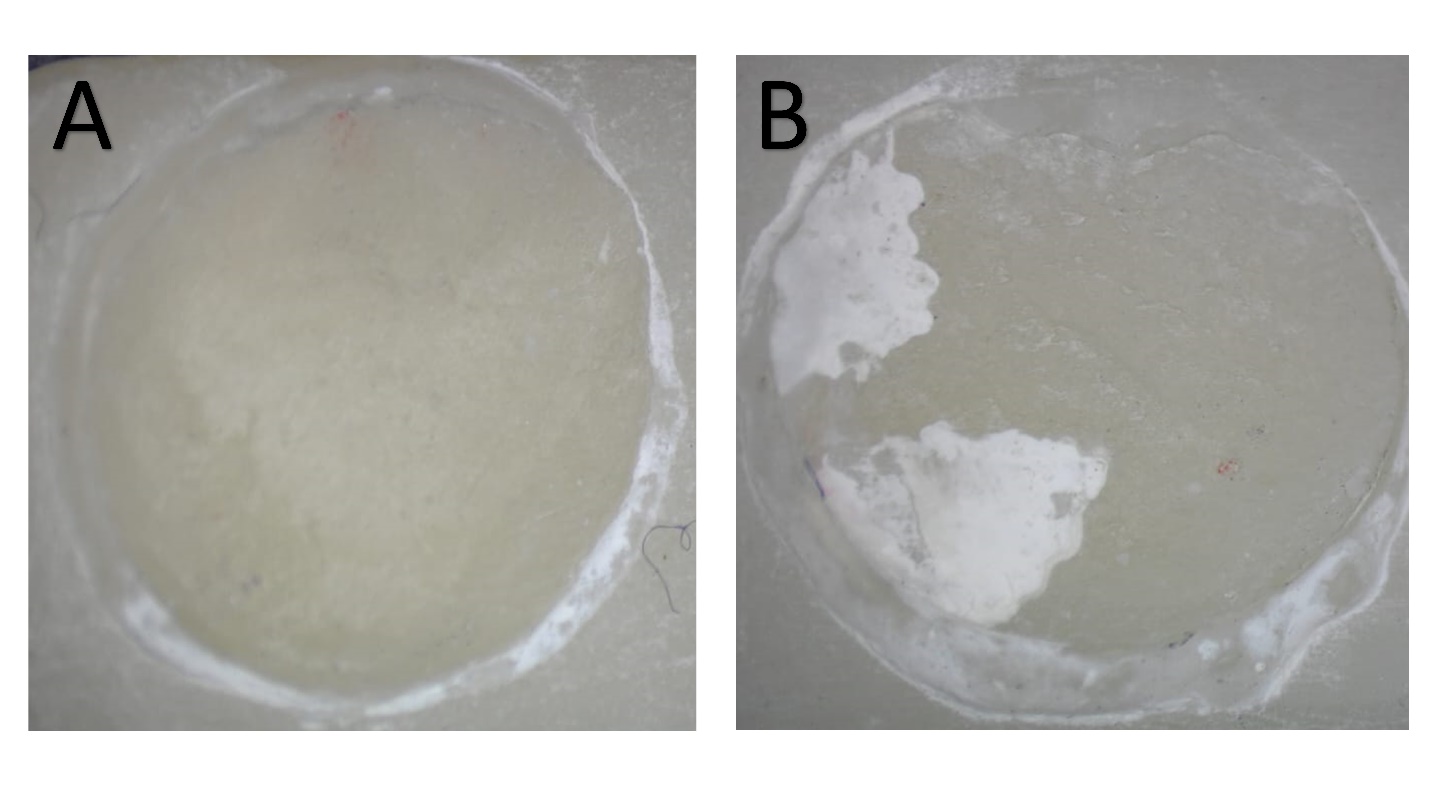
**

Supplementary file - [*Figure 1: Sample of faliure mode analysis. (A) Adhesive faliure. (B) Mixed faliure.*](#_Toc120997086)
